# Organoids and organs-on-chips: insights into predicting the efficacy of systemic treatment in colorectal cancer

**DOI:** 10.1038/s41420-023-01354-9

**Published:** 2023-02-22

**Authors:** Jialong Zhu, Linlin Ji, Yitian Chen, Huiyu Li, Mengxi Huang, Zhe Dai, Jing Wang, Dan Xiang, Gongbo Fu, Zengjie Lei, Xiaoyuan Chu

**Affiliations:** 1 Department of Medical Oncology, Jinling Hospital, The First School of Clinical Medicine, Southern Medical University, Nanjing, 210000 China; 2 Department of Medical Oncology, Affiliated Jinling Hospital, Medical School, Nanjing University, Nanjing, 210000 China; 3 Department of Medical Oncology, Jinling Hospital, Nanjing Medical University, Nanjing, 210000 China; 4 Department of Medical Oncology, Jinling Hospital, Nanjing University of Chinese Medicine, Nanjing, 210000 China

**Keywords:** Tissue engineering, Cancer models

## Abstract

Cancer heterogeneity has posed a great challenge to traditional cancer treatment, with the reappearance of cancer heterogeneity of inter and intra patients being especially critical. Based on this, personalized therapy has emerged as significant research focus in recent and even future years. Cancer-related therapeutic models are developing, including cell lines, patient-derived xenografts, organoids, etc. Organoids are three-dimensional in vitro models emerged in the past dozen years and are able to reproduce the cellular and molecular composition of the original tumor. These advantages demonstrate the great potential for patient-derived organoids to develop personalized anticancer therapies, including preclinical drug screening and the prediction of patient treatment response. The impact of microenvironment on cancer treatment cannot be underestimated, and the remodeling of microenvironment also allows organoids to interact with other technologies, among which organs-on-chips is a representative one. This review highlights the use of organoids and organs-on-chips as complementary reference tools in treating colorectal cancer from the perspective of clinical efficacy predictability. We also discuss the limitations of both techniques and how they complement each other well.

## Facts

- Tumor treatment needs personalized treatment based on standardized treatment.
- Organoid can recapitulate the characteristics of parental tumor.
- Several organoid studies have proved that organoid has great potential to predict drug response.
- Organ-on-chip can simulate microenvironment by combining two or more cell types.
- The combination of organoid and organ chip can provide broad research prospects for precise tumor treatment.

## Open questions

- How to carry out effective clinical transformation of drug prediction function of organoid model?
- How to effectively combine organoid and organ-on-chip?
- How can the interaction of these two models be used for the study of immunotherapy and antivascular therapy?

## Introduction

Colorectal cancer (CRC) now ranks third in estimated new cases and emerges in the top three leading causes of death [1, 2]. There are proposals to bring forward CRC screening from 50 to 45, implying that cancer risk becomes earlier [3, 4]. Although advanced surgical techniques and modified adjuvant therapy have resulted in good colorectal cancer treatment outcomes, yet, CRC mortality remains high due to metastasis and post-treatment recurrence [5]. Colorectal cancer is a group of heterogeneous neoplastic diseases that usually originate from abnormal crypts. The heterogeneity of CRC can show distinct clinical and pathological features, leading to diverse outcomes and prognoses. Most colorectal cancers show changes in proto-oncogenes and tumor suppressor genes [6]. Generally, it is the result of an accumulation of genetic changes and epigenetic modifications over time and shows abnormalities in some of the following signaling pathways: Wnt/β-catenin, EGFR-RAS-RAF, MEK-MAPK, PI3K, p53, and TGF-β-SMADs [6, 7]. These molecular biomarkers can be used as a predictive and prognostic tool based on the application of next-generation sequencing.

**Fig. 1:**
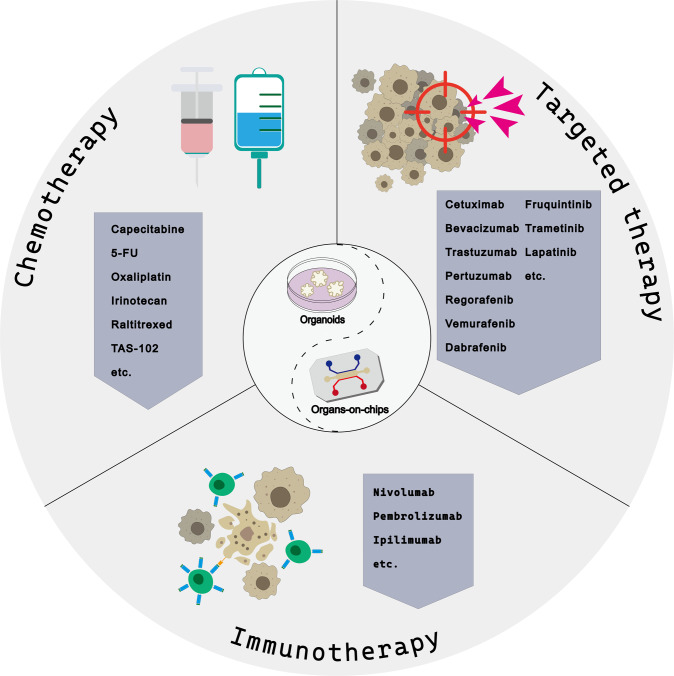
Systemic therapy for colorectal cancer. This figure depicts chemotherapy drugs, targeted drugs, and immunotherapy drugs commonly used in basic and clinical research of colorectal cancer based on organoid and organ chips.

The differences in clinical patient responses may be partly due to tumor heterogeneity. Thus, personalized treatment for different patients with different conditions is necessary, such as gene detection or evidence-based medicine [8]. Relying solely on diagnostic guidelines and molecular sequencing is insufficient to cover all patients. Human cancer cell lines are the most commonly used basic cancer and drug research models. However, few of them can fully recapitulate the mutation and transcriptional heterogeneity of primary tumors [9]. Another in vitro model, patient-derived xenograft (PDX), is relatively difficult to construct but with higher accuracy. The apparent limitations of its clinical application include low fluxes and long processing time. Because of the integrity of PDX microenvironment, it remains the gold standard for verifying drug sensitivity [10]. Organoids are three-dimensional cellular complex made by the self-organization of stem cells based on developmental biology principles [11], while organs-on-chips are microfluidic cell culture devices manufactured by the microchip manufacturing method [12]. The emergence of cancer organoids and organs-on-chips is currently suitable as a preclinical model for clinical transformation and precision medicine [13, 14] (Fig. 1). Significant advances in organoids and organs-on-chips technologies have facilitated the construction of in vitro near-physiological three-dimensional tissues and organs. Incorporating organoid precise recurrence of tumor features and organ-chip microenvironment integration may provide a tremendous opportunity to accelerate clinical transformation [15].

In this review, we discuss two preclinical models of the adult stem cell-derived organoid system and organs-on-chips technology efficacy in predicting systemic treatment of colorectal cancer. We focused on the consistency of in vitro and in vivo drug responses based on these two models and the feasibility of adjusting treatment strategies in clinical patients based on this predictive effect as a supplement to the clinical treatment reference index. Finally, we discuss the two models’ limitations and look forward to organoids-on-a-chip technology.

## The organoid system

Organoid is a three-dimensional cellular complex with a certain spatial structure formed by cells differentiated from stem cells cultured in vitro. Hans Clevers’s group in 2009 demonstrated that one single leucine-rich repeat-containing G protein-coupled receptor 5 (LGR5^+^) intestinal stem cell could initiate 3D crypt-villus organoids. The small intestine organoids cultures were suspended in Matrigel accompanied by R- spondin 1 (a ligand of LGR5 and WNT agonist [16], epidermal growth factor (EGF), and the bone morphogenetic protein (BMP) inhibitor noggin). To date, organoids from most parts of the human body have been established, including healthy and tumor organoids from brain [17, 18], esophagus [19], lung [20, 21], breast [22], liver [23, 24], stomach [25, 26], pancreas [27, 28], kidney [29, 30], colon [21, 31], bladder [32, 33], prostate [34, 35] and so on. The culture systems for colorectal cancer organoids are summarized in Table 1, while the articles without clear reporting methods have been excluded. Adult stem cells and induced pluripotent stem cells (iPSCs) are the two main sources of organoids. Higher cell integrity and a greater diversity of cell types are frequently observed in iPSC-derived organoids. Organoids can capture the characteristics of their corresponding parental tumors [36]. The histological and genetic characteristics between organoids and parental tumors are highly consistent. Even in the course of culture for a certain period, the corresponding characteristics are not lost [37].

**Table 1:** Culture systems for colorectal cancer organoids.

Cancer lesion	Enzyme	Concentration	Digestion time	Growth factors	Required or absent	Function of growth factors in organoid culture	Ref
Colon	Collagenase IX Dispase II	75 U/mL 125 g/mL	30–60 min	Adv.DF12 + + + Wnt3a* R-spondin Noggin EGF Nicotinamide N-acetylcysteine Gastrin-1 A83-01 SB202190 Y-27632 PGE2# FGF-2# FGF-10# N2 supplement# B27 supplement	+ +/− + + + + + + + + +/− +/− +/− +/− +/− +	Basal medium to reduce serum use Stemness/Wnt signaling activator Stemness/Wnt signaling enhancer BMP inhibitor Mitogen Stemness/cystic phenotype Antioxidant Mitogen/Prolong survival TGF-β inhibitor p38MAPK inhibitor ROCK inhibitor /Block apoptosis Wnt activator/cystic phenotype Mitogen Mitogen Cofactor mixture Cofactor mixture based on N2	[21]
Colon and Rectum	Collagenase II Hyaluronidase	1.5 mg/ml 20 ug/ml	30 min				[68]
Colon and Rectum, Liver and ovarian (metastasis)	Liberase TH TrypLE Express	1x 1x	60 min				[31]
Colon and Rectum	Collagenase IV Trypsin-EDTA	250 U/mL 0.05%	unknown				[10]
Colon and Rectum (metastasis)	PBS/EDTA TrypLE Express	1 mM 2x	60 min				[67]
Colon and Rectum, Liver and Peritoneum (metastasis)	Collagenase II Hyaluronidase IV	1.5 mg/mL 10 mg/mL	15–60 min				[100]
Rectum	Collagenase XI Dispase II TrypLE Express DNase I	100 U/ml 125 μg/ ml 1x 3 mg	40 + 10 min				[69]
Colon and Rectum	Collagenase A Hyaluronidase	0.5 mg/mL 20 mg/mL	30 min				[85]
Rectum	Collagenase IV Collagenase II Hyaluronidase Dispase II	500 U/mL 1.5 mg/mL 20 mg/mL 0.1 mg/mL	30–60 min				[70]
Peritoneum (metastasis)	Collagenase IV Dispase Hyaluronidase DNase I	67.5 U/mL 0.23 U/mL 8–20 U/mL 50 units/mL	30–60 min				[74]

Adv.DF12 + + + is Advanced DMEM/F12-based medium supplemented with HEPES, Glutamax, penicillin and streptomycin.

^*^Wnt3a is usually withdrawn to reduce normal organoid contamination in colorectal tumor organoid culture.

+, Required; −, Absent; # Not strictly required.

Furthermore, organoids outline many developmental-related biological parameters, such as heterogeneous cell construction and cell-cell/cell-matrix interactions. Current applications for cancer biology in tumor organoids mainly include screening for clinical and preclinical drugs [38] and drug discovery [39], the study of the exploration microenvironment [40] and tumor heterogeneity [22], as well as response prediction and personalized medicine [10]. Peculiarly, considering time cost, economic cost, operability, and success rate, the organoid system maintains particular superiority in drug response prediction and personalized medicine. Despite the potential of organoid models in biomedicine, there are significant limitations. Lack of a vascular, nervous or immune system is a major disadvantage of organoids. Besides, matrigel and other animal-derived matrices are commonly used in organoid cultures. Uncertain protein composition and variation among batches of animal-derived matrices are ongoing issues which may result in low controllability of cell microenvironment and low reproducibility of organoids. These constraints may prevent organoids from responding to specific pathophysiological parameters and carrying out downstream conversion application.

## Organs-on-chips

Organs-on-chips can be defined as a small functional unit that mimics the level of human organs in vitro [41]. Generally, it is based on an organ’s anatomy and simplified for assembly in vitro with the presence of essential elements necessary for physiological function [12]. Briefly, organ chips are microfluidic cell culture devices made of optically transparent materials, such as polydimethylsiloxane (PDMS). The main structure is the irrigation-controlled microchannel for the growth of living cells like cell lines, primary cells, and stem cells. The chips can simulate a microenvironment by combining two or more cell types. Different cells, such as organ-specific epithelial cells and stromal cells, are typically separated by ECM gel in different channels. Accordingly, it can recapitulate the multicellular structure of human organs and tissue-tissue interfaces, chemical gradients, vascular perfusion systems, and mechanical properties at the level of multicellular structure. At present, organ chips that encompass many organ types have been developed, including lung alveoli [42, 43] and bronchioles [44, 45], kidney [46, 47], liver [48], pancreas [49, 50], heart [51, 52], bone and bone marrow [53, 54] as well as the blood-brain barrier (BBB) [55, 56]. Some orthotopic cancer organ chips are developed to mimic tumor structure and physiology, such as lung adenocarcinoma chip [57], breast cancer chip [58] and multiple myeloma chip [59]. Table 2 summarizes the characteristics of CRC-related devices that have been reported in the articles since 2016 [60, 61]. The ability to capture human physiology and pathophysiology from a different perspective than organoids make them capable in vitro models for preclinical evaluation [62]. The dynamic study of microenvironment, the reduction of using animal models, novel drug development, drug efficacy, and toxicity assessment are all areas of applied research in organ chips technology that are currently in full swing [63].

**Table 2:** Technological overview of organ-on-a-chip models of colorectal cancer.

In vitro platform	Cancer cell type	Other biological components	Material	Chip configuration	Fabrication technique	Media exchange method	Ref
Vascularized micro-organs	HCT116, SW620, SW480	ECs, matrix and stromal cells	PDMS	2-layered channels	Soft lithography	Direct exchange	[77]
Organotypic tumor spheroids	MC38, CT26, Primary cells from human and mouse (Organotypic Tumor Spheroids)	Organotypic Tumor Spheroids contains immune cells and stromal cells	PDMS	3-layered channels	Soft lithography	Perfusion	[66]
Colorectal tumor chip	HCT116	HCoMEC	PDMS	3-layered channels	Soft lithography	Perfusion	[86]
CRC-on-chip	Caco2, C2BBe1, HCT116, HT29, CRC organoid	HUVEC and human primary fibroblasts	PDMS	2-layered channels	Soft lithography	Perfusion	[60]
Vascularized micro-tumors	HCT116, SW480	ECs, matrix and fibroblasts	PDMS	3-layered channels	Soft lithography	Direct exchange	[78]
Tumor-on-chip	SW620	Spheroids within Matrigel	Polycarbonate	2-layered channels	Micromachining	Perfusion	[61]

*PDMS* polydimethylsiloxane, *ECs* endothelial cells, *HCoMEC* human colonic microvascular endothelial cells, *HUVEC* human umbilical vein endothelial cells.

## Pre-clinical models for chemotherapy

Chemotherapy, surgery, and radiation are still considered the mainstream antitumor therapy for CRC in clinical setups and have improved many patients’ overall survival [5]. Despite its severe side effects and pervasive drug resistance, chemotherapy remains the most compelling part of systematic therapy. The chemotherapy backbone for CRC or mCRC treatment is drugs like 5-fluorouracil, capecitabine, oxaliplatin, and irinotecan, and several protocols that combine with them, such as FOLFOX, FOLRIRI, and CAPEOX regimen [64, 65] (Fig. 1). However, the chemotherapeutics strategies are constantly adjusted in response to changes in the patient’s actual condition. Colorectal cancer has entered the era of precision therapy, such as MMR/MSI status to predict the efficacy of adjuvant chemotherapy. Currently, preclinical models like organoids and organs-on-chips show great potential in anticancer-drug screening, personalized therapy, and drug response prediction associated with patient outcomes [66, 67]. These high-fidelity, high operational in vitro models have come to the fore in translational research.

Large-scale drug screening was performed in CRC and mCRC organoids in clinical trials and practice, including chemotherapeutic drugs and targeted agents [67, 68]. The results demonstrated the feasibility of high-throughput drug screening and forecasting the response of patients’ drug therapy. Based on these successful organoid-drug response platforms, a joint analysis of clinical patients’ therapeutic response and patient-derived organoids’ drug treatment response can sufficiently provide robust evidence [67]. For instance, they established a biorepository of 65 patient-derived rectal cancer (RC) organoids from lesions of primary, metastatic and recurrent; Ganesh and colleagues treated 21 different RC organoids with single-drug 5-FU and FOLFOX regimen (5-FU, leucovorin, and oxaliplatin). Based on the sufficient clinical follow-up of seven patients, the organoid drug responses significantly correlated with the corresponding patients’ progression-free survival [69].

Similarly, Yao and colleagues tested 80 rectal cancer organoids with single-agent 5-FU, irinotecan, or radiation and tested 23 with combinational therapies. Comparing monotherapy and combinational therapies in organoids almost remained consistent when they observed organoid drug responses with tumor regression grading (TRG) after surgical resection [70]. Although drug dosages employed during in vitro drug screening and in vivo, chemotherapeutics may remain significant, the sensitivity of the drugs could be reflected to verify the predictability of organoids. Lately, they have used liver metastatic organoids to complement their cohort and demonstrated that organoids have a good potential to predict drug sensitivity and clinical outcome of FOLFOX or FOLFIRI [71]. In addition to the single lesion, drug response on organoids from multiple lesions may explain the clinical reaction of specific patients. the test about TAS-102 (an oral combination agent) on mCRC organoid from different metastases in the same site (liver) and pre-and post-treatment metastases emphasized the correlation between heterogeneity and drug response as well as the consistency of efficacy [67]. The organoids from baseline segments were more sensitive to TAS102 than those from disease progression segments of pre-and post-treatment, consistent with the size of tumor foci from CT scan. Some prospective studies have provided the rudiment of preclinical guiding pharmacy. A successful chemotherapy response prediction was performed by mCRC organoids in contrast with CT and CEA tumor markers from the corresponding patient of treatment-refractory metastatic colon cancer along with alterations in APC and TP53. They received re-treatment of FOLFOX regimen and FOLFIRI regimen combined with panitumumab successively [72]. Before the patient benefitted from re-treatment of FOLFOX chemotherapy, organoids were confirmed sensitive to 5-FU and oxaliplatin but not to single-agent 5-FU.

Voest and colleagues reported another prospective clinical study to verify the feasibility and potential value of PDO as a predictive tool for chemotherapeutics regimens in CRC. Although there was no intervention with the patient, their PDO-based classifier indicated that it is clinically feasible to employ mCRC organoid to deliver a prediction on the outcome of irinotecan-based chemotherapy, yet, it failed on 5-FU-oxaliplatin combination therapy [73]. Still, with prospective studies that recruited 28 CRPMs patients, Woods and colleagues could not distinguish samples from 9 patients with partial response (PR) or stable disease (SD) versus progressive disease (PD) following FOLFOX treatment based on FOLFOX sensitivity of peritonoids [74]. The potential mechanism of the role of FOLFOX in mCRC organoids may be worth figuring out [75]. Impressively, they supplied a gemcitabine-capecitabine combination to a patient whose peritonoids were sensitive to multiple therapeutics. FDG PET-CT scans showed partial response after three months of treatment but followed by disease progression in further two months of treatment [74]. This variation in drug potency is prevalent in clinical settings, possibly due to multiple heterogeneous lesions or other unknown changes. Resampling organoids from altered lesions may aid in revealing changes in the tumor itself following treatment.

The organs-on-chips (OOCs) platform is another preclinical system for evaluating responses to cancer therapies. The combination of spheroid culture and microfluidic platform may be a successful strategy in the OOCs field to mimic the in vivo tumor microenvironment for drug screening of anticancer candidates [76]. Although low simulation of the tumor itself in cell lines or spheroids compared to organoids, the endotheliocyte, fibroblast, and immune cells can be assembled in organs-on-chips to mimic a relatively intact tumor microenvironment [77–79]. Earlier, the microfluidic devices combined with spheroids from human LS174T colon carcinoma cells were exposed to a continuous fluid medium to mimic microenvironment gradients in the vasculature of solid tumors [80]. A vascularized micro tumors platform was recently reported to test FOLFOX regimen and single agents such as 5-FU, oxaliplatin, and vincristine in three CRC cell lines and endothelial cells, matrix cells, stromal cells [77]. More recently, another vascularized micro tumor model (VMT) was also tested with FOLFOX regimen but compared with corresponding monolayer cultures, spheroids, and xenografts, and the tumor growth curves of VMT and xenografts were highly similar [78]. In reality, not many studies have been done to predict the anticancer agent efficacy of CRC on organs-on-chips [14]. Relatively ideal curative efficacy prediction requires primary culture spheroids or primary culture cells to ensure a degree of similarity to the patient’s parental tumor. In future studies, OOCs model may construct cellular components with primary culturing, microenvironment-related cells, and vasculature to evaluate drug response and mechanisms of drug action [66, 79]. Following that, drug concentrations similar to blood, chemical gradients caused by drug penetration, and drug response under microenvironment will be studied and adjusted as closely as possible to the drug response in vivo, based on dynamic perfusion of vasculature and integrated microenvironment. Chemotherapy efficacy can thus be predicted to some extent in this manner.

Organoids can be used as a therapeutic prediction tool and have been preliminarily used to instruct adjustment of patients’ chemotherapy strategies [71]. With the improvement of organs-on-chips technology, efficacy prediction can also be carried out from another aspect.

## Targeted therapy prediction for individual

The genomic and heterogeneous similarities between patient-derived tumor organoids and corresponding parental-tumor specimens have aided in developing personalized cancer therapy [81]. Compared to chemotherapy, targeted therapy is more dependent on gene-drug associations. The well-known biologic anti-EGFR agents, such as cetuximab, and panitumumab, consistently execute poor efficacy for RAS and RAF mutant CRC tumors, leading to the indispensability of testing KRAS and NRAS, and BRAF mutations before considering the anti-EGFR therapies [5, 68] (Fig. 1). The two main signaling pathways of EGFR activation are the RAS-RAF-MAPK pathway and PI3K-PTEN/PTEN/AKT pathway. Many other targeted drugs in clinical practice have been derived from these two pathways, such as BRAF-inhibitors, MEK inhibitors, etc. Another renowned biologic agent was the anti-VEGF monoclonal antibody like bevacizumab, aflibercept, and ramucirumab. Unfortunately, resistance to targeted drugs is as common as chemotherapy drugs. Some KRAS and BRAF wild-type CRC patients also could not benefit from anti-EGFR agents because of the effect from other relevant pathways and potential pathway crosstalk [82]. The detection of molecular characteristics alone is no longer sufficient for the existing clinical adjustment of clinical therapeutic strategy [10]. Compared to potentially complex resistance mechanisms that would necessitate extensive research, combining a better phenotype and simple molecular characteristics would be a more direct and effective method.

**Fig. 2:**
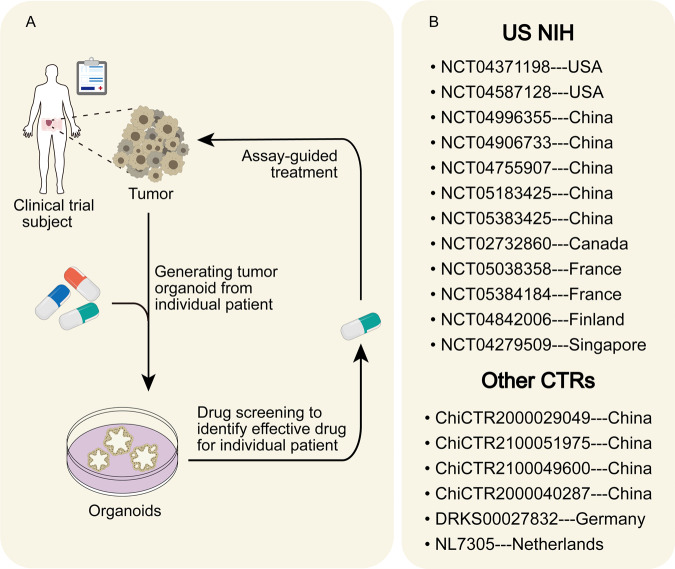
Organoids as a supplement of the clinical treatment reference index. **A** Patient-derived tumor organoids have the potential to choose therapy for the individual patient. It can provide feedback to the clinician to adjust the treatment decision based on the drug screening results. **B** This shows the registration numbers of ongoing clinical trials based on colorectal cancer organoids [129], including registered Clinical trials from US ClinicalTrials.gov, Chinese Clinical Trial Registry, etc [130, 131].

Organoid platforms were verified that allow for genomic and functional studies at the level of individual patients [68]. The gene-drug association captured by organoids lays the foundation for investigating the molecular basis of drug response and predicting targeted therapy efficacy [83]. Despite a lack of validation in xenograft models and patients, the initial gene-related drug screening on CRC organoids shows that KRAS mutant organoids and KRAS wild-type/BRAF mutant organoids were insensitive to cetuximab, which were consistent with the response of targeted clinical therapy [68, 84]. Some KRAS-mutated organoids could be restrained by combinatorial EGFR-KRAS G12C inhibition [85]. Ulteriorly, Two CRC cases were involved in combination drug screens. KARS/TP53 mutated organoid was insensitive to most drugs, including frequently-used 5-FU, oxaliplatin, and EGFR inhibitors, consistent with clinical patient response [84]. The trametinib-based combinatorial targeted treatment, on the other hand, was highly influential on this gene-type organoid but was not validated in PDX models [10]. Another APC-mutant organoid was more insensitive to the combination of afatinib and histone deacetylase (HDAC) inhibitors than FOLFOX regimens accepted by patients at the time [10]. The result was further verified in corresponding PDX models. Five PDOs and their respective mCRC patients have recently tested the predictive value to the response of EGFR blockage cetuximab. The most impressive was the biopsy, along with EGFR amplification as well as no RAS pathway mutation, from a disease-progressive patient who initially responded to cetuximab but gradually turned to resistance. No response to cetuximab showed in its organoids consistent with the patient’s clinical response [67]. Signally, multiple tyrosine kinase inhibitors, regorafenib, have also been consistent clinical responses in PDO-xenografts due to lacking vasculature in organoids [67]. This predictive value must eventually manifest in the patient. The attempts are made by two CRC peritonoids and their respective patients. The combination of MEK inhibitors trametinib and cobimetinib and the multi-tyrosine kinase inhibitor vandetanib significantly reduced the viability of KRAS G12D-mutant organoids that were resistant to most monotherapy chemotherapeutics. No obvious response was found with adjusted four weeks of vandetanib therapy in the patient but did not include MEK inhibitors [74]. The other peritonoid showed sensitivity to adavosertib, the combination of EGFR inhibitor Osimertinib, and HDAC inhibitor vorinostat. However, adavosertib remains in phase II clinical trial for advanced colorectal cancer, and two other drugs were restricted by funding and off-label use [74]. Received gemcitabine–capecitabine combination therapy ultimately according to drug access, cost, and toxicity; this patient displayed partial response (PR) but still turned to disease progression (PD) subsequently, as described before. The general pattern of transformation from organoid to clinical is displayed in Fig. 2a. The Netherlands Cancer Institute performed a prospective clinical study (SENSOR study, NL50400.031.14 EudraCT2014-003811-13) involving colon cancer and NSCLC patients for targeted treatment. In this study, patients’ treatment strategies would be altered with screening results from organoids of these patients’ tumors [81]. Other registration numbers for other organoid clinical trials are listed in Fig. 2b. The organoids may be particularly appropriate as predictive tools for targeted therapies, considering the robust capture of genomic alterations.

Organs-on-chips could also be used for screening specific drugs. The in vitro vascularized micro tumors (VMTs) are a relatively representative model in colorectal cancer. The vasculature derived from endothelial cells commendably complements the composition of microenvironment [77, 78]. Ten kinds of clinically-approved receptor tyrosine kinase (RTK) inhibitors targeted to VEGFR2, PDGFR, Tie2, and FGFR1, including pazopanib and sorafenib, were tested in VMTs [77]. Novel targeted drug like galunisertib, a TGF-βR1 antagonist, finished in phase II clinical trials for hepatocellular cancer, was also tested in VMT loading with SW480 and HCT116 attributed to the presence of fibroblasts in the microenvironment [78] In addition, drug delivery and penetration have been performed in similar colorectal cancer organ-chip systems [86, 87]. Though the fidelity of the tumor itself is not superior to the organoid, the integrity of microenvironment can better explore the interaction of medicine and the cell-extracellular matrix. The influence of complex microenvironments on drug efficacy cannot be ignored, such as drug targeting to the stroma, dynamic microenvironmental drug concentrations, and microenvironmental barriers [88]. These conditions can also provide a basis for predicting the efficacy of targeted therapy in organs-on-chips.

By assembling primary tumor cells, patient efficacy can be predicted to a certain extent. Similar efficacy prediction studies have been conducted on other tumor chips. Two chips were combined to test the anti-ES tumor effect and cardiotoxicity of lincitinib, a selective IGF-1R inhibitor. The results were also compared with those from clinical trials [89]. Notably, Hickman and colleagues established a reconfigurable multi-organ system to investigate anticancer drug efficacy and off-target effects in two different cancer-derived models. One was the combination of two leukemia cell lines and primary hepatocytes to study imatinib and diclofenac. Another system used to study tamoxifen was a multidrug-resistant vulva cancer line, a non–multidrug-resistant breast cancer line, primary hepatocytes, and induced pluripotent stem cell-derived cardiomyocytes [90]. The results were compared to published clinical and preclinical data. These cases provide direction and gist to explore drug efficacy prediction and evaluation in the organs-on-chips system, even in colorectal cancer.

## Development of personalized immunotherapy

Immunotherapy, including immune checkpoint inhibitors and adoptive cell therapy, regulates the tumor microenvironment or immune system to identify and attack tumor cells [91]. Unfortunately, immunotherapy plays a limited role in solid tumors, while yields unusually brilliant results in hematologic tumors. For 4–5% of CRC with mismatch-repair-deficient (dMMR) or high levels of microsatellite instability (MSI-H), immune checkpoint inhibitors (ICIs), including PD-1 blockade and CTLA-4 inhibitor, have received US Food and Drug Administration’s (US-FDA) approval [92, 93] (Fig. 1). ICIs, on the other hand, are ineffective in most clinical CRC patients with mismatch-repair-proficient (pMMR) and microsatellite-stable (MSS) or low levels of microsatellite instability (MSI-L) due to low mutant neoantigen burden and a lack of immune cell infiltration [94, 95]. Several MSI CRCs with a high mutational load could not benefit from ICIs [96]. In addition, adoptive T cell therapies have limited colorectal cancer effects. Unlike pharmacotherapy, the improvement of cell therapy always directly depends on many clinical studies subjected to many restrictions [97–99]. Developing cell therapies for solid tumors may therefore be slow. Accordingly, as a predictive tool, the emerging preclinical models may effectively accelerate the development of immunotherapy in solid tumors.

Organoids generally lack immune system components, making tumor-immune interaction difficult. Exploiting the air-liquid interface (ALI) system for the immune microenvironment and organoids/immune cell co-culture system makes it possible to study immunotherapy [100, 101]. ALI system could retain the original innate immune components for a certain period. Another one was an artificial combination of organoids and immune cells. The co-culture system of peripheral blood lymphocytes and tumor organoids were validated primarily in dMMR patients. In four of eight major histocompatibility complex (MHC) class I + colorectal organoids, CD8^+^ T cells showed tumor-specific responses but not in MHC class I deficient organoids [100]. MHC class I deficiency was also inherited from its parental tumor. Numerous studies have found that HLA/MHC gene expression is closely related to ICI treatment response [102, 103]. The co-culture result showed that tumor-responsive T cell populations could be amplified and the capture of MHC-based immune reactivity. The author and colleagues co-cultured organoids with T cell populations loading tumor responsive with or without MHC class I and MHC class II blockages. The cytotoxic effect of T cells was observed in CRC organoids but not in healthy organoids [100]. General stratification of patient immunotherapy response includes expression of the immune checkpoint, Infiltration of immune cells, mismatch repair mechanism, tumor neoantigen burden [104], TCR clonality, and immune gene signatures. These factors have more or less been studied and recapitulated in CRC organoids, containing the expression of HLA molecular [100–103, 105].

The capture of immune-related components and co-culture system testifies to the possibility of ICIs efficacy prediction. Apparently, this treatment prediction requires validation from more prospective studies and clinical outcome correlation to screening appropriate patients for enriching clinical ICIs response. Voest and Haane reported an ongoing exploratory NICHE study about neoadjuvant immunotherapy in pMMR and dMMR early-stage colon cancer [106]. 100% and 27% responses were found in patients with dMMR and pMMR colon cancer, respectively, after the employment of CTLA-4 inhibitor and PD-1 blockage for neoadjuvant treatment. Notably, they investigate the low reactivity of pMMR tumors in clinical responders’ co­culture platforms. The reactivity of autologous T cells in an organoid co-culture system was limited to colon tumors that responded clinically to ICIs. In vitro reactivity was not observed in all clinical responders [106]. The result proves the predictability of organoids in ICI therapy from the side. Otherwise, Farin and colleagues developed a quantitative co-culture platform for CAR-mediated cytotoxicity toward PDCOs. They proved that organoids could be tested for CAR toxicity in a complex and competitive microenvironment by co-culture of EGFR variant III-expressing CRC organoids, EGFRvIII-CAR NK-92 cells, and healthy colon organoids [107]. Likewise, the application of organoid-based adoptive cell therapy required further exploration in vivo experiments [100, 107]. Compared to chemotherapy and targeted therapy, the prediction of personalized immunotherapy responses in CRC organoids response suffer more challenges.

A popular organ-on-chip type is similar to the organoid-immune cell co-culture system but with a higher degree of integration. Cancer cell lines, cancer-associated fibroblasts (CAFs), immune cells from healthy donors’ PBMCs, and endothelial cells are typically involved in this system. Within the chips, dynamic changes in the tumor immune microenvironment can be quantified and visualized [108]. Accordingly, some mutual problems may be figured out. For instance, trastuzumab may promote cancer-immune cell interactions and CAFs played an inhibitory role in the immune microenvironment in breast cancer [79]. Whereas the lack of individual characteristics is insufficient for personalized precision immunotherapy. The main part of organs-on-chip construction needs to come from individual patients to accomplish the prediction of immunotherapy [66]. The murine- and patient-derived organotypic tumor spheroids (MDOTS/PDOTS) are more satisfactory subjects for forecasting immunoreaction. It retained autologous lymphoid and myeloid cell populations [66, 109]. This type of OOCs was proved that could simulate the response to PD-1 blockade and ascertain specific interventions to counteract resistance [66]. However, it was limited to tumor infiltrating lymphocytes (TILs) and could not reflect the recruitment of additional immune cells [109]. The evaluation of immune cell recruitment has also been carried out in Breast tumor-on-a-Chips [110]. The addition of autologous circulating immune cells or biomimetic lymph nodes may be a future direction [111, 112]. Even though organs-on-chips have some advantages in the integration of immune-related systems, there is still a lack of research closely related to clinical transformation.

## Future prospects

Further exploration of these in vitro preclinical models has revealed many limitations. Compared to cancer cell lines, patient-derived organoids and organs-on-chips consume more time and resources in culturing. The primary limitations of tumor organoid culture are the lack of a microenvironment, the short-term expansion, contamination from normal organoids, the effects of serum and growth factors in the culture system, and the requirement for mouse-derived extracellular matrix (ECM). Meanwhile, despite simulating a relatively complete microenvironment, organs-on-chips are limited to technical robustness, time cost, raw materials for chips, and relatively lower complexity of 3d structure than organoids. Many of them have the potential to influence the prediction of curative effects. For example, except for organoids derived from colorectal cancer, some organoid drug efficacy comparisons for other cancers have not been particularly favorable [113], which may directly relate to the lack of microenvironment [114]. Likewise, the same is valid for fetal bovine serum and some mouse-derived small molecules or ECM [115, 116]. Routinely, effective predictive models require drug screening in the window of meaningful short-term clinical treatment. Long-term response to drug treatment is also critical. In terms of immunotherapy, the long-term clinical response has been unsatisfactory, attributed to immune failure related to the immune microenvironment. In the model, this may necessitate extending the duration of drug action, analyzing the internal changes of individual immune cells [117] and constantly introducing new lymphocytes or forming multi-organ organ chips containing lymph nodes to stimulate immune cell recruitment from tertiary lymphoid structure [111]. The presence of a small number of potentially healthy organoids or deliberately importing healthy organoids may make the outcome of drug response more precise [100, 107]. This demonstrates that in vitro model has great potential for improvement. Thommen and his colleagues built a patient-derived tumor fragment platform to dissect the early immunological response to ex vivo PD-1 blockade. Compared to matched clinical response data, the ex vivo immunological response clinical outcomes were in concordance with clinical outcomes in a high degree of 26 patients, including 12 melanoma and non-small cell lung cancer (NSCLC) patients who did not respond to clinical PD-1 blockade [118]. This gives inspiration to microenvironment capture. It also shows the importance of early immunotherapy intervention [106] and of tracking the long-term dynamics of the immune microenvironment from the side. Furthermore, low cost and high throughput must be considered a reference supplement for individual clinical efficacy prediction. This role could be filled by commercial organs-on-chips [88]. Therefore, without limitation to the perfection of the model itself, being consideration of pros and cons of these two models, organoids-on-a-chip may be a promising future direction.

Organoids-on-a-chip is an integration of organoids with organ-on-a-chip technology. It’s essentially an interaction between the stem cell field and the engineering field. Organoids are further designed to break the limitations by controlling stem cell behavior and cell microenvironment. Organs-on-chips are the target organ’s artificial construction and are precisely controlled artificially [119]. Organoids follow intrinsic developmental programs to self-assemble [11]. The fusion of these two distinct but complementary technologies has been accepted by most scientists [81, 120, 121]. Enhancing fidelity and reproducibility is the top priority task of organoids-on-a-chip [15]. The biophysical and biochemical microenvironment and the nutrient supply must be controlled during the assembly of organoids-on-a-chip.

**Fig. 3:**
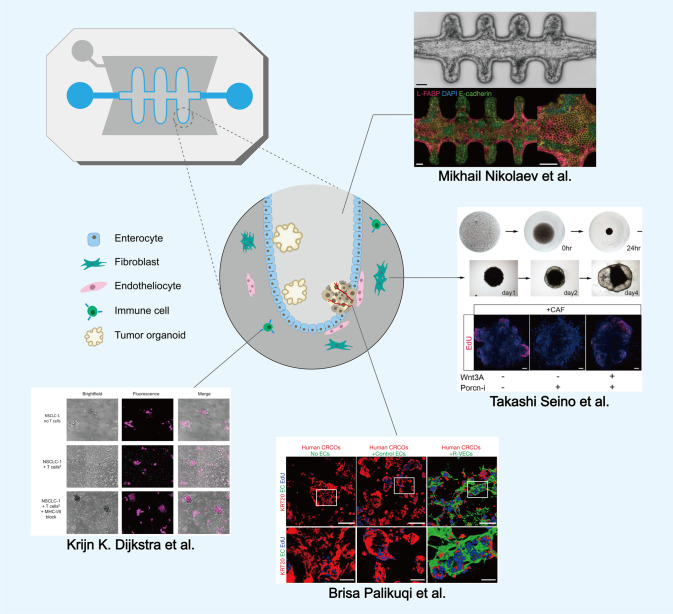
Organoids meet organs-on-chips to perfect the quasi-physiological structure. The figure depicts a possible future, more potent in vitro technology by loading organoids and other stromal cells into microfluidic chips to complement the limitation of the two models. One channel was loaded with cancer organoids and enterocytes, while the other was coated with stromal cells. The middle figure is based on Ref. [126]. The top right is adapted from Ref. [126], Springer Nature Limited. The middle right is adapted from Ref. [116], Cell Limited. The lower one is adapted from Ref. [125], Springer Nature Limited. The lower left one is adapted from Ref. [100], Cell Limited. The surrounding experimental figures do not represent the actual situation of this model.

Mechanical flow has been shown to promote additional physiological integrity in developing PSC-derived intestinal and islet organoids [122, 123]. Physiological flow promoting angiogenesis was demonstrated on kidney organoids-on-chip [124]. An embryonic-like malleable vasculogenic endothelial cells was reported can break through the limitations of synthetic scaffolds and semi-permeable membranes with its ability to self-organize 3D lumenized vascular networks. Co-cultures of organoids with the specialized endothelial cells can serve as a tissue-specific biological platform containing vascular niche [125]. Analogously, The co-culture system of cancer-associated fibroblasts and pancreatic cancer organoids has also been applied to niche studies [116]. Lutolf and colleagues constructed an intestinal organoids-on-a-chip to attain more physiologically relevant organ shapes, sizes, and functions [126]. The application of fluid scour, and lumen structure scaffold well simulates the biophysical microenvironment. Remarkable tissue homeostasis was shown in the self-organization of functional intestinal epithelium in confining hydrogel scaffold. Induced epithelial injury and long-term parasite infection verified the regenerative and physiologic mimicry of intestinal organoids-on-a-chip. The authors also introduced intestinal myofibroblasts and macrophages to mimic organ-level complexity [126]. Lutolf’s team recently developed a hydrogel-based microfabrication method for controlling the spatiotemporal morphogenesis of intestinal organoids with defined shape and structure [127]. Morphogenesis of other tissues may be extended based on this in the future. These strategies further improve organoid and organ-on-chip models in a visual and controllable manner, allowing the possibility of reproducing in vivo complex tissues or organs in vitro. High fidelity may be achieved by introducing a complete microenvironment, including the vasculature, and its application may take developmental medicine and regenerative medicine to a new level. We can consider the possibility of organoid-on-hips with personalized characteristics of patients based on these analogous models (Fig. 3). The patient’s primary cells are introduced into the chip in an organoid fashion, and various types of stromal cells are also delivered to further refine the physiological structure in vitro. The dynamic changes in drug response can then be detected in the flow system.On the one hand, it increases fidelity to complete the current bottleneck in basic research. On the other hand, it simplifies the technical difficulty within a controllable range to improve repeatability to serve as more versatile and predictive preclinical tools for precision medicine [15, 128]. This synergistic engineering may be a pivotal bridge to translation from lab to clinic.

## Conclusion

Although it remains a long way from being practical and widely used, it has great potential in predicting therapeutic efficacy. A corresponding number of clinical trials based on these models are currently underway. In terms of the treatment time window, cost, drug screening efficiency, and accuracy, these in vitro models will likely be used as a clinical reference shortly to guide the optimization of treatment strategies for individual patients through technological advancement or technology fusion.
